# Acute Effect of Repeated Sprint Exercise With Blood Flow Restriction During Rest Periods on Muscle Oxygenation

**DOI:** 10.3389/fphys.2021.665383

**Published:** 2021-07-29

**Authors:** Chihiro Kojima, Keiichi Yamaguchi, Hiroto Ito, Nobukazu Kasai, Olivier Girard, Kazushige Goto

**Affiliations:** ^1^Japan Institute of Sports Sciences, Tokyo, Japan; ^2^Graduate School of Sport and Health Science, Ritsumeikan University, Shiga, Japan; ^3^School of Human Sciences (Exercise and Sport Science), The University of Western Australia, Crawly, WA, Australia

**Keywords:** local hypoxia, occlusion, perfusion, recovery, repeated sprint ability

## Abstract

**Purpose:**

This study aimed to examine the effect of applying BFR during rest periods of repeated cycling sprints on muscle oxygenation.

**Methods:**

Seven active males performed 5 × 10-s maximal pedaling efforts with 40-s passive rest, with or without BFR application during rest period. BFR was applied for 30 s between sprints (between 5 and 35 s into rest) through a pneumatic pressure cuff inflated at 140 mmHg. *Vastus lateralis* muscle oxygenation was monitored using near-infrared spectroscopy. In addition, blood lactate concentration and heart rate were also evaluated.

**Results:**

The BFR trial showed significantly lower oxyhemoglobin (oxy-Hb) and tissue saturation (StO_2_) levels than the CON trial (*P* < 0.05). However, power output and blood lactate concentration did not significantly differ between the two trials (*P* > 0.05).

**Conclusion:**

Applying BFR during rest periods of repeated cycling sprints decreased muscle oxygenation of active musculature, without interfering with power output during sprints.

## Introduction

Repeated sprint exercise (RSE), consisting of several bouts of maximal sprints (<10 s) separated with relatively short rest periods (<60 s), has been shown to increase maximal oxygen uptake (VO_2m__*ax*_), repeated sprint ability (Bishop et al., 2011) and improve muscle buffer capacity (Bishop et al., 2004). It is therefore not surprising that this training modality is popular in various sport activities such as soccer, lacrosse, and rugby. One of the most important training stimuli for maximizing the effects of RSE is metabolite (e.g., lactate and hydrogen ion) accumulations within working muscles (Bishop et al., 2004). A single bout of RSE can markedly increase muscle lactate content and decrease muscle glycogen content (Gaitanos et al., 1993). Because both metabolite production and clearance are both closely associated with local blood flow in surrounding muscle tissues, restricted blood flow (e.g., attenuated blood supply to muscles or blood outflow from muscles) would enhance metabolite accumulation.

Sprint interval exercise (SIE), consisting of repetition of several “all-out” efforts of longer duration (>20–30 s) separated with several minutes of rest periods, combined with post-sprint blood flow restriction (BFR) has been already reported (Taylor et al., 2016; Mitchell et al., 2019). For instance, BFR during rest period between sprints of SIE augmented hypoxia-inducible factor-1α (HIF-1α) (Taylor et al., 2016). The previous study indicated the possibility to activate HIF-1α-mediated signaling involved in angiogenesis induced by greater tissue hypoxia when combining SIE with BFR (Taylor et al., 2016). On the other hand, the effect of RSE with or without continuous (sprints + rest periods) BFR of upper limb or lower limb (thigh) muscles throughout the session (sprints + rest periods) on muscle oxygenation has been recently investigated (Willis et al., 2018, 2019b,c; Peyrard et al., 2019). Although the addition of BFR during repeated sprinting lowers the tissue saturation index (as a proxy for local hypoxia severity; Willis et al., 2019a), power output during the actual sprints efforts was attenuated (Peyrard et al., 2019). The attenuated power output during sprint exercise with BFR may be associated with the ability of the neuromuscular system to produce maximal force (Peyrard et al., 2019) and/or a relationship between force and velocity during repeated sprinting (Fenn and Marsh, 1935; Davies et al., 1984). Furthermore, phosphocreatine (PCr) degradation/resynthesis and aerobic metabolism might be partially involved (Gaitanos et al., 1993). Since the oxygen delivery to promote resynthesis of muscle PCr during rest periods would be insufficient due to BFR, detailed information of muscle oxygenation during RSE with BFR is likely to be informative. On the other hand, there was no studies which focused on the influences of BFR during rest periods of RSE on sprint performance and muscle oxygenation. It is possible (but unknown) that the application of BFR during the rest periods between sprints only may augment deoxygenation levels (larger training stimulus), yet without interrupting with power production (maintained training quality) in reference to a control trial during the actual efforts of a repeated sprint cycling exercise.

Therefore, the purpose of the present study was to test the hypothesis that the addition of BFR during rest periods of a repeated cycling sprint exercise would accentuate deoxygenation levels, while fatigue resistance during the actual sprint bouts will not be compromised.

## Materials and Methods

### Participants

Seven active males volunteers [age (mean ± standard deviation, SD): 21.7 ± 0.8 years, height: 172.2 ± 5.4 cm, body weight: 65.9 ± 4.7 cm, body mass index: 22.2 ± 1.6 kg/m^2^] belonging to the same lacrosse team, took part in this study. Participants were informed of the purpose, experimental procedure, and potential risk of this study, and written informed consent was obtained. This study was approved by the Ethics Committees of Ritsumeikan University, Japan.

### Experimental Overview

The present study was designed using a randomized cross-over design. All participants completed RSE either with BFR (BFR trial) or without BFR (CON trial) during rest periods between sprints on different days. Each trial was separated with at least 2 days. Muscle oxygenation, power output and heart rate were recorded during the exercise. Blood lactate concentrations were evaluated before and after exercise.

### Experimental Protocol

**FIGURE 1:**
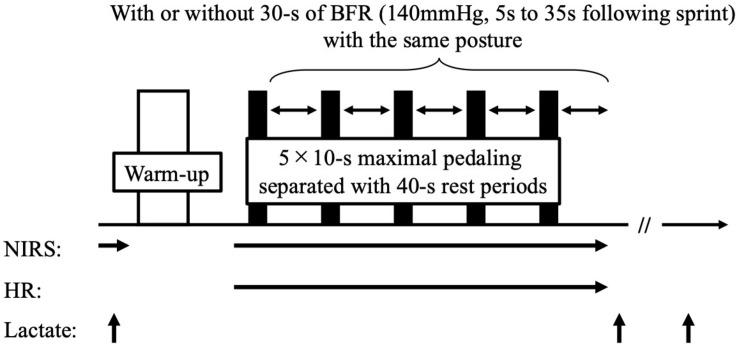
Experimental protocol.

Participants were instructed to replicate the last training and meal prior to the experiment and arrived at laboratory following their general daily training (14:00–15:00). Following sufficient rest (around 20 min), baseline muscle oxygenation and blood lactate concentration were evaluated. After the baseline measurements, participants performed warm-up (2 × 3 s maximal pedaling exercise with the load of equivalent to 5.0 and 7.5% of body weight, respectively) followed by RSE (5 × 10 s maximal pedaling exercise, separated by a 40 s rest between sprints) using an electromagnetically braked cycling ergometer (Power Max VIII; Konami Corporation, Tokyo, Japan) with or without BFR during rest periods (Figure 1). The load during the pedaling was set as equivalent to 7.5% of body weight. During the entire RSE, BFR cuffs (Rapid version cuff, Hokanson Inc., Bellevue, WA, United States) were placed bilaterally to the most proximal part of each leg in both trials. In the BFR trial, the cuffs were inflated to 140 mmHg for 30 s between sprints (between 5 and 35 s into rest) in each leg. When pressure was applied, participants maintained an upright posture by dropping the right leg while sitting on the bike. The pressure and duration for BFR were determined from previous studies (Taylor et al., 2016; Mitchell et al., 2019) and several our preliminary studies to confirm whether the exercise is possible to complete without profound power reduction and pain. During CON trial, the cuffs remained deflated (without pressure) while participants were requested to maintain an identical posture to in the BFR trial. Immediately and at 5 min after completing the fifth BFR (35 s later following completion of the exercise), blood lactate concentrations were measured.

### Measurements

#### Muscle Oxygenation Evaluated by Near-Infrared Spectroscopy (NIRS)

Uninterrupted (sprints and rest periods) measurements of the muscle oxygenation of the right *vastus lateralis* were obtained using a portable NIRS system (Hb14-2; ASTEM Co., Ltd., Kanagawa, Japan). The probe was attached to the skin surface emits two different wavelengths from near-infrared light (wave length: 770 and 830 nm), with average optrode–detector distances of 20 and 30 mm, respectively, and the penetration light (depth below the skin surface; 100 and 150 mm) is detected in the light-receiving element. The probe was placed on the right thigh, at the middle point between the great trochanter and knee joint (*vastus lateralis* muscle) and covered with a black cloth to protect it from light. The oxy-Hb, deoxy-Hb, and total-hemoglobin (total-Hb) levels were recorded at a sampling frequency of 10 Hz and were averaged during each sprint and rest period. The values were expressed relative to pre-exercise values while the participant was in the sitting position for 1 min.

#### Blood Lactate Concentration

Blood samples were collected from a fingertip to evaluate blood lactate concentrations before, immediately (35 s later due to fifth BFR) after exercise and at 5 min following fifth BFR. Blood lactate concentrations were measured immediately after collecting blood samples using lactate analyzer (Lactate Pro, ARKRAY Co., Kyoto, Japan).

#### Heart Rate

Heart rate was recorded continuously every second throughout the exercise using heart rate monitor (RCX5, Polar Electro Oy, Finland). Also, heart rate was averaged during every sprint (10 s) and rest period (40 s) and used for further analysis.

### Statistical Analyses

Data are expressed as means ± SD. Normal distribution was confirmed using Kolmogorov-Smirnov test for all variables. A two-way repeated-measure ANOVA was used to determine the main effects of trial, time, and trial × time interaction. If significant effects were found, a Tukey-Kramer *post-hoc* test was used for pairwise comparison. Power output was compared between trials using a paired *t*-test. Statistical significance was determined at *P* < 0.05. Effect sizes (ES) were calculated by partial eta squared (η^2^) for two-way ANOVA with repeated measures.

## Results

**FIGURE 2:**
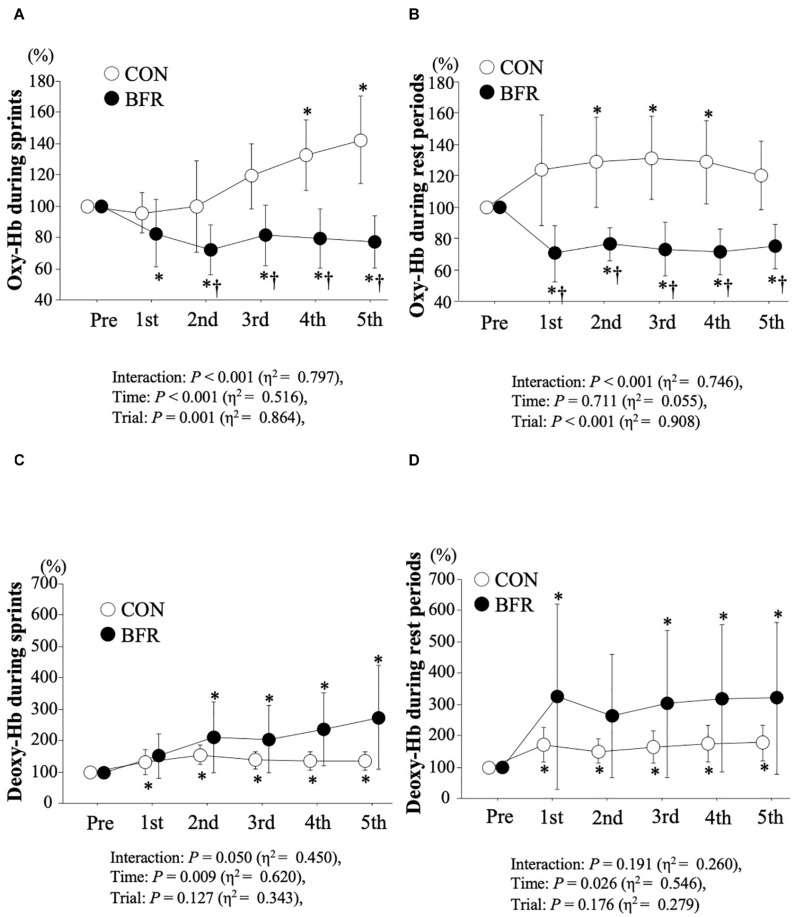
Oxy-Hb levels (*top panel*; **(A,B)**, respectively) and deoxy-Hb levels (*bottom panel*; **(C,D)**, respectively) during sprints and rest periods. Values are means ± SD. ^∗^*P* < 0.05 vs. Pre, ^†^*P* < 0.05 vs. CON.

Figure 2 showed time-course changes in oxy-Hb and deoxy-Hb levels. Statistical analysis revealed a significant trial × time interaction and main effects of time and trial for oxy-Hb during both sprints (interaction: *P* < 0.001, η^2^ = 0.797, time: *P* < 0.001, η^2^ = 0.516, trial: *P* = 0.001, η^2^ = 0.864) and rest periods (interaction: *P* < 0.001, η^2^ = 0.746, time: *P* = 0.711, η^2^ = 0.055, trial: *P* < 0.001, η^2^ = 0.908). Oxy-Hb levels were significantly lowered in the BFR trial than CON trial during sprint and rest periods (*P* < 0.05). A significant main effect for time was found in deoxy-Hb during sprints (*P* = 0.009, η^2^ = 0.620) and rest periods (*P* = 0.026, η^2^ = 0.546). In contrast, there was a tendency of interaction (*P* = 0.050) for deoxy-Hb during sprints, but the ES was modest (η^2^ = 0.450).

**FIGURE 3:**
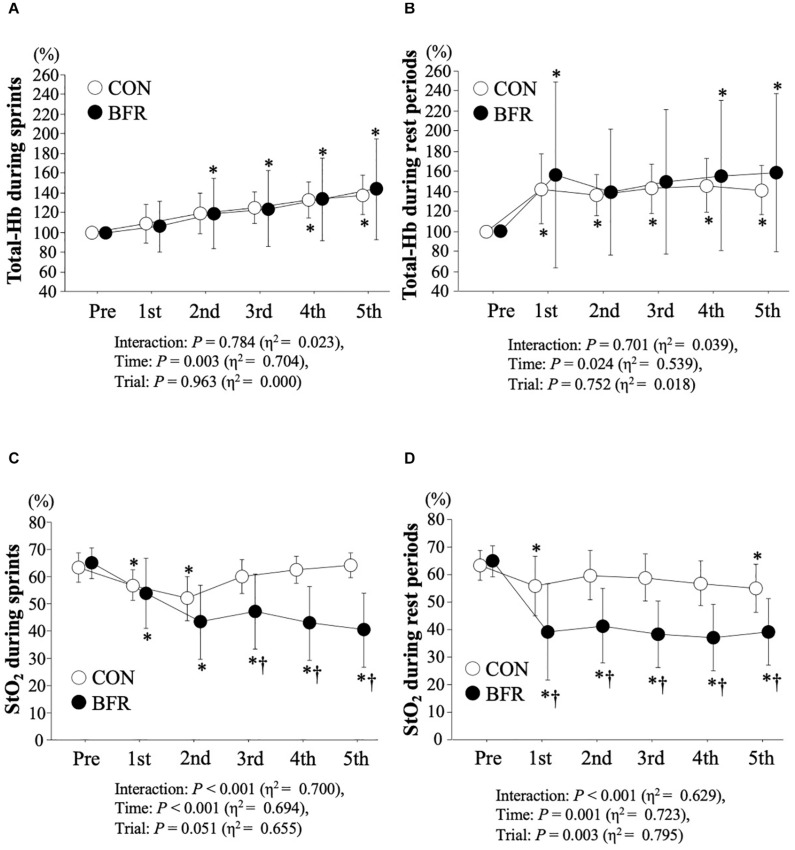
Total-Hb levels (*top panel*; **(A,B)**, respectively) and StO_2_ levels (*bottom panel*; **(C,D)**, respectively) during sprints and rest periods. Values are means ± SD. ^∗^*P* < 0.05 vs. Pre, ^†^*P* < 0.05 vs. CON.

Time-course changes in total-Hb and StO_2_ levels were indicated in Figure 3. A significant main effect of time was detected in total-Hb during sprints (*P* = 0.003, η^2^ = 0.704) and rest periods (*P* = 0.024, η^2^ = 0.539). Two-way ANOVA reveled that a significant interaction, main effect for time and/or trial in StO_2_ during sprints (interaction: *P* < 0.001, η^2^ = 0.700, time: *P* < 0.001, η^2^ = 0.694) and rest periods (interaction: *P* < 0.001, η^2^ = 0.629, time: *P* = 0.001, η^2^ = 0.723, trial: P = 0.003, η^2^ = 0.795). StO_2_ levels were significantly decreased throughout the exercise with BFR with significant lower values in BFR trial than in CON trial after the third set of sprints and rest periods between all sprints (*P* < 0.05).

**FIGURE 4:**
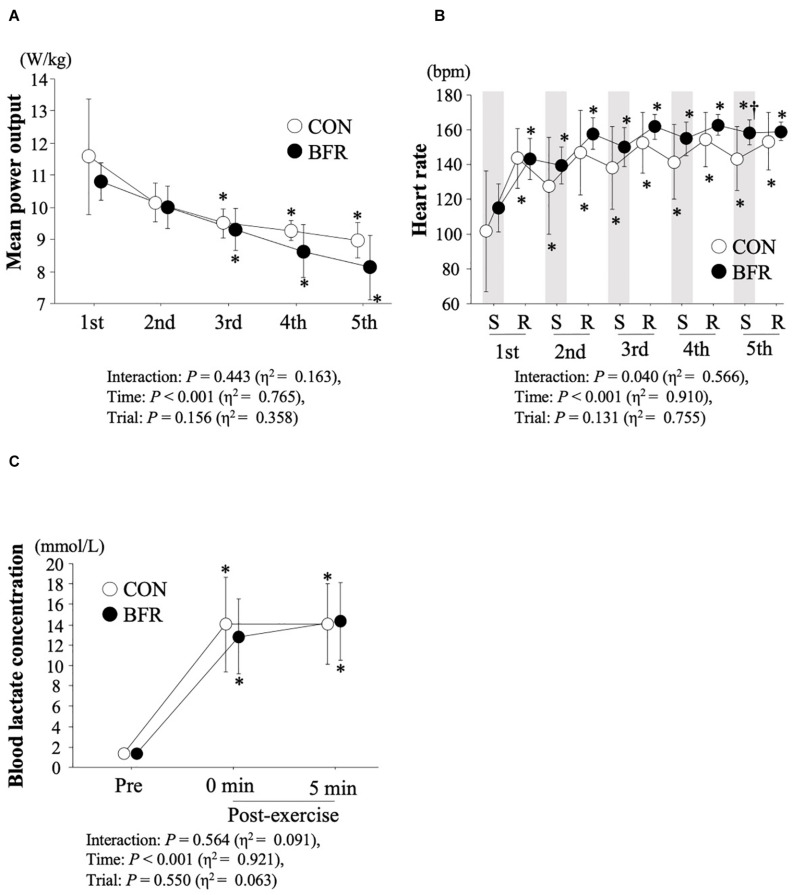
Mean power putout **(A)**, heart rate **(B)** during sprints and rest periods, and lactate concentrations before and after exercise **(C)**. Values are means ± SD. ^∗^*P* < 0.05 vs. Pre, ^†^*P* < 0.05 vs. CON. S, sprint; R, rest period. Gray bar indicates average heart rate during sprint exercise in each set.

Figure 4 indicated time-course changes in mean power output, heart rate throughout the exercise and blood lactate concentrations immediately and at 5 min after exercise. Statistical analysis revealed a significant main effect for time in mean power output (*P* < 0.001, η^2^ = 0.765). Mean power output was significantly reduced from the third to fifth set of sprints in both trials. However, there was no significant difference between trials (*P* > 0.05). In addition, the total power output (sum of power output over first to fifth sets of sprints) did not differ between BFR and CON trials (46.8 ± 2.9 W/kg vs. 49.5 ± 2.4 W/kg for five sprints, *P* = 0.16). When the peak power output was calculated, a significant interaction (*P* = 0.001, η^2^ = 0.574) and main effect of time (*P* = 0.001, η^2^ = 0.861) and trial (*P* = 0.014, η^2^ = 0.735) were observed. Peak power output was significantly decreased with progress of sprints in both trials (*P* < 0.05), and significantly lower values were observed in BFR trial than those in CON trial at fourth and fifth set of sprints (*P* < 0.05). A significant interaction (*P* = 0.040, η^2^ = 0.566) and main effect for time (*P* < 0.001, η^2^ = 0.910) were observed in time-course changes in heart rate. Heart rate was significantly increased with progress of sprints in both trials, and significantly higher value was observed in BFR trial than that in CON trial during fifth set of sprint (*P* < 0.05). For blood lactate concentrations, there was a significant main effect of time (*P* < 0.001, η^2^ = 0.921). Blood lactate concentrations were significantly increased with exercise and significantly higher values were also observed even at 5 min after exercise in both trials.

## Discussion

The purpose of the present study was to investigate the acute effects of repeated sprints with or without BFR during rest periods between sprints on muscle oxygenation. Our main finding was that both oxy-Hb and StO_2_ levels (but not deoxy-Hb and total-Hb levels) were significantly lower when BFR was applied during rest periods, despite mean power output throughout the exercise was similar between trials. Therefore, the application of BFR during rest periods of a RSE could be an efficient strategy for producing sustained local hypoxia in working muscles, without compromising power production.

In the present study, both oxy-Hb and StO_2_ levels were lower when BFR was applied during rest periods. Generally, decreased oxy-Hb with increased deoxy-Hb are observed during the actual sprint across repetition, before rapidly returning near baseline during rest periods between efforts (Racinais et al., 2007; Smith and Billaut, 2010). Therefore, it appears that BFR during rest periods interferes with reoxygenation in working muscles. However, total-Hb, a marker of blood volume (Van Beekvelt et al., 2001), remained unchanged between trial at any time point. A recent study demonstrated that total-Hb levels during repeated double-poling sprint exercise were significantly higher under systemic hypoxia trials than under normoxia, probably due to augmented vasodilation via nitric oxide production (Yamaguchi et al., 2019). Furthermore, there was no significant difference in time-course changes in deoxy-Hb levels between trials. Christiansen et al. (2018) indicated that BFR during sprint interval running exercise significantly increased deoxy-Hb levels. In the present study, BFR was applied only during 30-s rest periods between sprints, and with same pressure among participants, suggesting that relative pressure was possible to be different for among participants. It is plausible that the duration of BFR and/or how to apply the pressure (140 mmHg), which would affect the severity of local hypoxia, was insufficient to alter total-Hb and deoxy-Hb levels.

Most previous studies (Fujita et al., 2007; Abe et al., 2010; Kim et al., 2014) have focused on the impact of BFR application during the actual exercise period (e.g., resistance or endurance exercise). Additionally, these BFR studies commonly utilized low-intensity exercise with BFR, since the application of cuff pressure during high-intensity exercise would not successfully restrict blood circulation within working muscles due to augmented muscle pump action. However, two previous studies succeeded to determine the combined effects of SIE (30 s maximal efforts with several minute of rest periods) with BFR (∼130 mmHg) during rest periods (Taylor et al., 2016; Mitchell et al., 2019). Actually, Taylor et al. (2016) investigated gene expression in working muscles following a single bout of SIE (repeated 30-s maximal sprints) with post-exercise BFR application during the first 2 min into the 4.5 min of rest periods between efforts. These authors observed that HIF-1α mRNA expression was significantly augmented following a single bout of SIT when post-exercise BFR was applied, suggesting that tissue was exposed to hypoxia and greater metabolic stress was produced with 2 min of BFR between sprints (Taylor et al., 2016). Unlike these previous studies, the present study is the first to directly report local hypoxia (decreased oxy-Hb and StO_2_ levels within working muscles) induced by brief (30 s) periods of BFR after each sprint during an acute RSE session using NIRS. On the other hand, Peyrard et al. (2019) and Willis et al. (2019b, c) determined the effect of BFR throughout entire leg or arm RSE sessions on muscle oxygenation. In these studies, BFR was maintained during both sprints and rest periods, which was different from the present study. These authors indicated that the addition of BFR to RSE consequently augmented blood perfusion (Willis et al., 2019a), but that power output during arm sprint with BFR was significantly attenuated (Peyrard et al., 2019). In contrast, the application of BFR between sprints in the present study had no negative impact on power output. Our data indicate, for the first time, it is likely that blood flow restricted during rests of repeated cycling sprints would allow the working muscles to exert local hypoxia without attenuating power output across sprints repetition.

However, we did not find significant difference between trials in blood lactate concentrations, suggesting that metabolic perturbations might not be augmented in BFR trial. Future studies are necessary to evaluate intramuscular lactate concentration and metabolic acidosis (e.g., blood hydrogen ion, bicarbonate ion, and base excess) to clarify the impact of the present training strategy on metabolic regulations. In addition, peak power output was attenuated during the latter phase of exercise with BFR although there was no significant difference between trials in mean power output and total power output throughout the sprints. Several previous studies demonstrated that repeated sprint performance was associated with PCr resynthesis involving in blood flow and aerobic metabolism (Gaitanos et al., 1993; Bogdanis et al., 1996; Dawson et al., 1997) as well as glycolysis system. The present study demonstrated that Oxy-Hb and StO_2_ levels were lowered in BFR trial, suggesting that PCr content might not be fully resynthesized due to BFR during rest period between sprints at the latter phase of sprints. Therefore, determinations of PCr kinetics during interest rest periods of RSE with BFR, in addition to muscle oxygenation, would be required in future studies. Furthermore, the comparison of acute physiological response and training adaptations between “local hypoxia (i.e., BFR)” and “systemic hypoxia (i.e., normobaric hypoxia)” would be a valuable challenge. A recent review demonstrated that a single session of strenuous exercise with BFR produced augmented physiological stimulus for vascular resistance and altered vasodilatory responses compared with systemic hypoxia during the same exercise (Willis et al., 2019a).

In conclusion, an acute RSE session combined with BFR during rest periods between sprints decreased oxy-Hb and StO_2_ levels within working muscles, but did not affect power output, deoxy-Hb, or total-Hb. This represents a meaningful intervention for augmented internal stress (training stimulus) without negatively impacting training workload (training quality).

## Data Availability Statement

The raw data supporting the conclusions of this article will be made available by the authors, without undue reservation, to any qualified researcher.

## Ethics Statement

The studies involving human participants were reviewed and approved by the Ethics Committees of Ritsumeikan University. The patients/participants provided their written informed consent to participate in this study.

## Author Contributions

CK and KG designed the present study. CK, KY, HI, NK, and KG collected and analyzed the data. CK, OG, and KG undertook the data interpretation and manuscript preparation. All authors approved the final version of the manuscript.

## Conflict of Interest

The authors declare that the research was conducted in the absence of any commercial or financial relationships that could be construed as a potential conflict of interest.

## Publisher’s Note

All claims expressed in this article are solely those of the authors and do not necessarily represent those of their affiliated organizations, or those of the publisher, the editors and the reviewers. Any product that may be evaluated in this article, or claim that may be made by its manufacturer, is not guaranteed or endorsed by the publisher.
